# I remember it now, so I’ll remember it later: Working memory strength guides predictions for long-term memory performance

**DOI:** 10.3758/s13421-023-01514-3

**Published:** 2024-03-25

**Authors:** Julia Krasnoff, Alessandra S. Souza

**Affiliations:** 1 Department of Cognitive Psychology, University of Zurich, Binzmuehlestrasse 14/22, 8050 Zurich, Switzerland; 2 Center for Psychology, Faculty of Psychology and Education Sciences, University of Porto, Porto, Portugal

**Keywords:** Judgments of learning, Working memory, Memory-strength theory, Cue-utilization approach

## Abstract

Judgments of learning (JOLs) are assumed to be made inferentially, based on cues. This cue-utilization approach substituted the theory that memory strength guides JOLs. The rejection of this theory ignores the existence of two memory systems: working memory (WM), which holds representations immediately accessible, and long-term memory (LTM), which is a permanent store. By manipulating and measuring WM strength, we tested a revised version of the memory-strength theory in which JOLs are guided by WM representations. In Experiment 1, participants memorized sequences of two or four colored objects, then they provided JOLs for an LTM test of these objects, and performed a WM test on the objects’ colors. After learning 200 objects, the LTM test followed. Sequence-length affected WM, but not LTM performance. JOLs, however, were higher for sequences of two than for four objects and correlated higher with WM than LTM performance. We replicated these results with a simultaneous presentation of the objects (Experiment 2), in the absence of a WM test (Experiment 3), and in a word-pair task (Experiment 4). Overall, our findings are consistent with the revised memory-strength theory. WM strength should therefore be considered when examining the factors guiding JOLs.

The ability to accurately monitor one’s learning enables people to allocate their study time more strategically (Metcalfe & Finn, 2008; Metcalfe & Kornell, 2005) and thus contributes to people’s learning success. A central aspect of learning monitoring is the ability to make accurate predictions of future memory performance, so-called judgments of learning (JOLs). Making those JOLs requires people to predict their long-term memory performance based on the limited information that is available at the time point when JOLs are made—for example, during learning. In the past decades, a growing body of research has examined which information guides JOLs. Two theories have been proposed: The *cue-utilization approach* (Koriat, 1997) and the *memory-strength* or *direct-access theory* (see Arbuckle & Cuddy, 1969; Hart, 1965). Whereas the cue-utilization theory has increasingly gained popularity, the memory-strength theory has fallen out of favor and is considered outdated. In the present work, we argue that the main arguments for the rejection of the memory-strength theory are problematic, and we propose a revised version of this theory—namely, that people use the strength of their working memory representations to guide predictions of their long-term memory performance. In four experiments, we provide evidence that the strength of working memory representations guides JOLs.

## Cue-utilization approach and the memory-strength theory of JOLs

The cue-utilization approach (Koriat, 1997) assumes that people do not have direct access to their memory strength. Instead, they rely on a variety of different cues to infer their likelihood of retrieving certain information in the future. Those cues can be intrinsic characteristics of the study items (e.g., word frequency), extrinsic characteristics of the study situation (e.g., presentation duration), but also mnemonic cues such as the ease people feel when processing the items (e.g., retrieval fluency). The utilized cues change depending on the time point when JOLs are made, and they can be more or less predictive for future memory performance, explaining why JOLs are sometimes inaccurate (Bjork et al., 2013). Since the cue-utilization idea was proposed, many studies focused on identifying potential cues. In those studies, participants are commonly asked to memorize items (e.g., lists of words or word pairs) for a long-term memory test. Certain properties of the study items or the learning situation (e.g., word frequency or presentation duration) are varied at encoding in two or more experimental conditions. The goal of this procedure is to test whether people use those properties as cues to make JOLs. In the most common version of the procedure, JOLs are requested immediately^1^ after the presentation of one study item (e.g., a word or word pair). Finally, after studying all items, participants perform a memory test (e.g., free recall or cued recall). JOLs are then aggregated as a function of the experimental condition. A difference in JOLs between the conditions is interpreted as evidence for the use of this property as a cue for JOLs. To examine whether the cues are diagnostic, long-term memory test performance is also compared between the conditions. If JOLs and test performance vary similarly as a function of the experimental condition, the manipulated cue is considered as *valid*. In contrast, if JOLs and test performance are unrelated, the use of the cue is considered a *metacognitive illusion* (Benjamin et al., 1998; Koriat & Bjork, 2005; Yan et al., 2016). Using this procedure, studies identified several cues such as word frequency (Fan et al., 2021), semantic relatedness of word pairs (Matvey et al., 2006; Mueller et al., 2013; Rhodes & Castel, 2008), font size (Mueller et al., 2014; Rhodes & Castel, 2008; Undorf & Zimdahl, 2019), word loudness (Rhodes & Castel, 2009), number of study items (Tauber & Rhodes, 2010), retrieval fluency, and encoding fluency (Hertzog et al., 2003; Koriat & Ma’ayan, 2005; Undorf & Erdfelder, 2011, 2013). Whereas most identified cues are predictive for future memory performance, some are not related to future performance and thus lead to wrong memory predictions (Rhodes & Castel, 2008, 2009).

In contrast to the cue-utilization theory that assumes that JOLs are inferential in nature, the memory-strength theory assumes that people directly access their memory strength and base their JOLs on it (see Arbuckle & Cuddy, 1969; Hart, 1965). A key implication of this theory is that factors that influence JOL do so because they have a direct impact on memory strength and hence on performance. Koriat (1997) first discarded this memory-strength hypothesis arguing that it could not explain why some experimental manipulations affect JOLs but not delayed memory performance (and vice versa). In contrast, numerous studies showed that certain manipulations affect participants’ JOLs and delayed performance in different ways (Koriat, 1997; Rhodes, 2016). To date, these findings, together with the ample number of studies on the use of cues, are taken as main evidence against the memory-strength theory.

## Reasons for revisiting the memory-strength theory for JOLs

There are four main reasons why we consider the rejection of the memory-strength theory as premature. First, Koriat’s argument only holds under the assumption that memory representations remain stable over time or, at least, degrade at a similar speed independent of the experimental manipulation. However, studies (Roediger & Karpicke, 2006a, 2006b) have shown that some manipulations (e.g., repeated study) boost performance when memory is tested after short delays (e.g., 5 min), but harm performance at longer delays (1-week). Conversely, other manipulations (e.g., testing memory) lead to worse performance at short delays, but increased performance at longer delays. Here, everyone would agree that performances at both time points depend on memory strength, although they are uncorrelated. A related and more germane critique is that this argument does not consider the involvement of two different memory systems: working memory, which maintains a small amount of information accessible in mind, and long-term memory, which is a more permanent memory store. JOLs are commonly made immediately after studying, and hence the study information is still maintained in working memory. JOLs are, however, compared with performance at a delayed test which requires retrieval of information from long-term memory instead of working memory. Working memory is capacity limited (Cowan, 2010), and thus sensitive to manipulations, such as memory load, that do not influence long-term memory (Bartsch, Loaiza, & Oberauer, 2019a, 2019b; Brady et al., 2013; Loaiza & Halse, 2019). Conversely, certain learning strategies (such as elaboration) were found to improve long-term memory but not working memory (Bartsch et al., 2018; Bartsch, Loaiza, Jäncke, et al., 2019a, 2019b; Bartsch & Oberauer, 2021). These findings show that working memory and long-term memory representations are relatively independent, although these systems may interact and contribute simultaneously to performance in many tasks (for a discussion, see Cowan, 2008). Hence, if participants are basing their JOLs on working memory representations that are uncorrelated with long-term memory representations, this could explain why JOLs often fail to accurately predict future performance. To test this revised memory strength theory for JOLs it is necessary to compare JOLs with memory performance at the time point when JOLs are made. This comparison has been neglected in previous literature.

The second reason to revisit the memory-strength theory is that most prior studies requested JOLs in situations in which working memory strength variation was artificially restricted. As mentioned above, items were often sequentially presented and JOLs were assessed directly after their offset (Mueller et al., 2014), hence the to-be-rated words were almost perfectly maintained in working memory when JOLs were assessed. The lack of memory-strength variance paired with the requirement to give different JOLs to different items could have nudged participants into using rather irrelevant cues that they would not consider in a natural study environment. To test the revised working memory strength theory for JOLs, it is crucial to implement at least some variation in working memory strength for the to-be judged items. In contrast, keeping memory strength at ceiling for the items while asking participants to give different JOLs to different items puts participants in a torn situation: They cannot comply with the instruction while at the same time relying on memory strength—even if this is what they would naturally do.

The third reason to revisit the memory-strength theory is the growing body of literature on metacognition in working memory that shows that people have access into their working memory and can accurately judge how well information is represented therein (Forsberg et al., 2021; Honig et al., 2020; Krasnoff & Oberauer, 2022; Rademaker et al., 2012; Suchow et al., 2016; van den Berg et al., 2017; Yoo et al., 2021). More recent studies on neural correlates of metacognition even suggest that uncertainty is directly maintained in working memory (Geurts et al., 2022; Li et al., 2021; Yoo et al., 2021). Hence, this literature shows that memory strength is not a variable that is inaccessible for metacognitive processes a priori. Even though these findings do not necessarily imply that people use this information to predict future memory performance, they raise the question of why people would focus on various cues if they can likewise just monitor their working memory strength to make an immediate JOL. This is especially questionable given that relying on cues would require maintaining them in working memory in addition to the study items which in turn would increase the working memory load during learning.

One could now oppose that the literature of the past decades already offered sufficient evidence for the use of cues, and thus reconsidering a revised version of the memory-strength theory would be an unnecessary setback. Most prior studies, however, did not measure and control for changes in working memory strength potentially evoked by the experimental manipulations. For instance, studies manipulating the semantic relatedness of word pairs found that JOLs vary with this manipulation (Matvey et al., 2006; Mueller et al., 2013). This finding was explained by the use of semantic relatedness as a cue for JOLs, even though it can be likewise attributed to increased working memory strength for semantically related as compared with unrelated word pairs. The latter memory-strength explanation is in line with ample evidence from the working memory literature reporting a strong working memory advantage for semantically related as compared with semantically unrelated lists (Kowialiewski et al., 2021, 2022, 2023). The same concern can be raised regarding several other findings that were interpreted as evidence for the cue-utilization theory without controlling for working memory effects: Either the utilized experimental manipulations of presumed cues have well-established effects on working memory strength (e.g., word frequency, Reder et al., 2016), or the effect on working memory strength has not been studied previously and therefore cannot be ruled out (e.g., font size). Table 1 presents a list of cues assumed to guide JOLs, whether they are predictive or not of delayed memory performance (LTM) and immediate performance (WM). As shown in Table 1, for several variables we see that JOLs go together with their effect on WM performance. Yet, for a number of manipulations, we have no data on how they impact WM. Accordingly, our fourth reason for further considering the memory-strength theory is that prior literature did not measure or control for immediate effects of the manipulated variables on memory strength. As a result, those findings do not invalidate the revised memory-strength theory we are proposing here. To ensure that differences in JOLs can be solely attributed to the use of cues and not to the use of working memory strength, controlling for effects of the manipulated variables on working memory is indispensable.

**Table 1:** Effects of manipulations on long-term memory (LTM), working memory (WM), and judgements of learning (JOLs)

Manipulation	Effect on:		
	LTM	WM	JOLs
Word frequency in free recall	↑ (Gregg et al., 1980)	↑ (Reder et al., 2016)	↑ (Fiacconi & Dollois, 2020)
Semantic relatedness	↑ (Matvey et al., 2006; Mueller et al., 2013)	↑ (Kowialiewski et al., 2021, 2022, 2023)	↑ (Matvey et al., 2006; Mueller et al., 2013)
Testing vs. restudying	↑ (Karpicke, 2009)	?	↓ (Karpicke, 2009)
Blocked learning vs. Interleaved learning	↓ (Dunlosky & Nelson, 1994; Krasnoff & Overkott, 2022)	↑ (Krasnoff & Overkott, 2022)	↑ (Dunlosky & Nelson, 1994; Krasnoff & Overkott, 2022)
Font-size	= (Kornell et al., 2011)	?	↑ (Kornell et al., 2011)
Perceptual interference	↓ (Besken & Mulligan, 2013)	?	↑ (Besken & Mulligan, 2013)
Identical word-pair (e.g., *cat–cat*) vs. related word-pair	= (Castel et al., 2007; Mueller et al., 2016)	?	↑ (Castel et al., 2007; Mueller et al., 2016)

↑ = positive association; ↓ = negative association

## The role of working memory for JOLs

Although working memory has not been measured in prior studies, the relevance of differentiating between working memory and long-term memory contributions to JOLs has been highlighted previously. Nelson and Dunlosky (1991), for instance, used it to explain the *delayed-JOL effect*: the well-replicated observation that JOLs are more accurate when solicitated with a delay (e.g., after the presentation of 10 intervening items) instead of immediately after item presentation. For example, in Nelson and Dunlosky’s study, the correlation of the delayed JOLs with recall was 0.9, whereas this correlation was only 0.38 for immediate JOLs. A meta-analysis of the delayed JOL effect (Rhodes & Tauber, 2011) has shown that JOL accuracy substantially increases irrespective of the size of the delay, which usually ranges from 1 min to 10 min. Nelson and Dunlosky (1991) hypothesized that delayed JOLs are more accurate (than immediate JOLs) because both delayed JOLs and delayed performance are related to the strength of long-term memory representations. Conversely, immediate JOLs are less accurate because they are contaminated by working memory traces. The results of more recent studies are consistent with this so-called monitoring dual-memories hypothesis. For instance, the accuracy of JOLs increases when they are preceded by a short distractor task (Bui et al., 2018; Kelemen & Weaver, 1997), which disrupts working memory maintenance. Contrarily, the accuracy of delayed JOLs decreases when the critical response (e.g., second word of word pair) is provided at JOL assessment (Dunlosky & Nelson, 1992), which activates this representation in working memory, obliterating long-term memory retrieval. Even though the relevance of the two memory systems has thus been discussed in previous literature, these findings are commonly not linked to the memory-strength theory. Instead, they are explained by the use of less predictive cues when providing immediate JOLs and more predictive cues when asked to make JOLs with a temporal delay (Koriat, 1997). The delayed JOL effect, however, can also be explained by direct access to memory traces: When JOLs are assessed immediately after studying (when the items are still in working memory), working memory strength is used as a basis for JOLs. When JOLs are assessed with a delay, long-term memory strength is used to make JOLs. Based on the memory-strength theory, the inaccuracy of immediate JOLs would thus result from the use of working memory representations that are not always diagnostic for future performance.

## The present study

In the present study, we provide a first test of the role of working memory strength in guiding JOLs. In four experiments using both visual and verbal materials, we manipulated working memory strength with a variation of memory load and compared JOLs with working memory and long-term memory performance. This manipulation allowed us to test predictions of the revised memory strength theory—but note that our study is silent about the adequacy of the cue-utilization hypothesis. The revised memory-strength hypothesis advanced here predicts that JOLs will vary with working memory strength. Hence, if we find that JOLs are independent of working memory performance, this provides conclusive evidence against the revised memory-strength hypothesis. If we do find that working memory strength guides JOLs, then this hypothesis can become a contender to explain JOLs that should be considered side-by-side with the prevalent inferential cue-utilization hypothesis in the future.

## Experiment 1

In Experiment 1, we manipulated working memory load when JOLs were requested to test whether working memory representations are used to make predictions about long-term memory performance. Unlike most studies examining JOLs that used verbal material (for a meta-analysis, see Double et al., 2018), we used an object-color continuous-reproduction task. In this task, participants are asked to memorize the color of various objects. At test, they are cued with the object and asked to reproduce its color on a color wheel. Whereas this material and task is novel to the field of metacognition, it is very commonly used in the literature on visual working memory (Prinzmetal et al., 1998; Souza et al., 2016; Zhang & Luck, 2008). This task has also been used to show that people have direct access to the quality of their working memory representations (Honig et al., 2020; Krasnoff & Oberauer, 2022; Mitchell & Cusack, 2018; van den Berg et al., 2017) and can use this information to guide decisions. Accordingly, we used it here to obtain a fine-grained measure of people’s memory performance allowing us to detect small variations in people’s memory strength and assess if this information would guide JOLs. We chose a manipulation of working memory load because prior work showed that this manipulation affects working memory fidelity but not long-term memory fidelity (Brady et al., 2013). If people, thus, rely on working memory representations to predict their future performance, JOLs should vary with working memory load and this variation should be uncorrelated with long-term memory performance.

### Method

#### Transparency and openness

Experiments 1 and 3 were preregistered. All data, analysis scripts, and materials to run the experiments are available in the project’s page at the Open Science Framework (https://osf.io/47wjp/).

#### Participants

We preregistered (https://osf.io/75dbw/?view_only=None) to collect a sample of 30 university students 18–35 years of age. The design of the experiment consisted of a within-subjects manipulation of memory load (two vs. four objects in a sequence). A previous study from our lab using the same visual materials found a memory load effect of *d* = 2.09 on immediate recall. To detect such a large effect with α = 0.05 and power = 95%, we would need a sample of only five participants. We decided to collect a sample-size six times larger because (a) we aimed to test whether the memory-load effect appears in the JOLs and delayed test, and (b) we initially aimed to fit the data with the visual Working Memory Confidence model (van den Berg et al., 2017). Students from the University of Zurich were recruited to take part in a lab-based study in exchange for 15 CHF or partial course credit in the year 2020. Participants were German-speaking individuals, and they self-reported no visual impairment. The following criteria for the exclusion of participants were preregistered: (a) Overall performance in the immediate recall task is not significantly different from chance level, (b) variance in JOLs is not different from zero, and (c) the experiment is not completed. Based on these criteria, no participants had to be excluded from the sample. The sample thus consisted of 30 participants (26 female, four male).

#### Materials and procedure

**Fig. 1:**
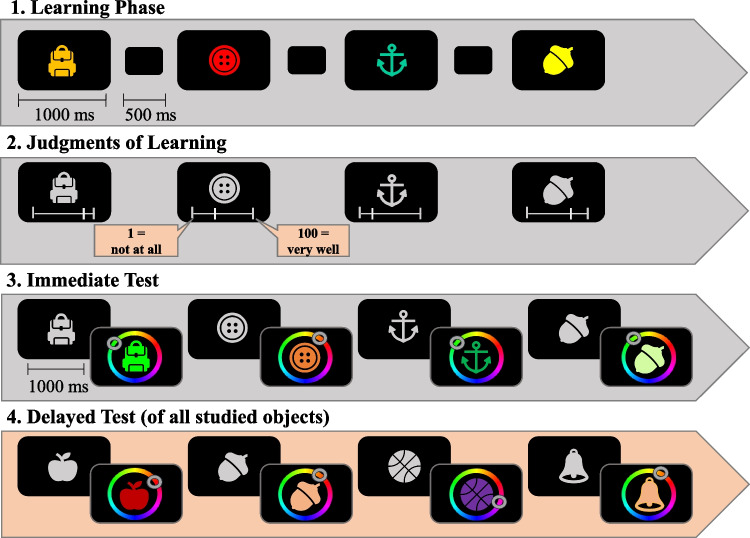
Experimental procedure of Experiment 1. *Note*. Flow of events of Experiment 1. Steps 1–3 depict a trial with a sequence-length of four (high working memory load). These steps repeated several times with randomly varying sequence-lengths. Step 4 was conducted only once after all sequences have been presented

The experiment was programmed in MATLAB using the Psychophysics Toolbox 3 extension (Brainard, 1997). The main experimental task consisted of an object-color continuous reproduction task. Objects were sampled from 321 clip-art images obtained from the set used by Sutterer and Awh (2016). For each participant, 200 objects were randomly selected from this pool to serve as memoranda in the experimental trials. Additionally, six objects were selected for two practice trials. Each object was randomly assigned one of 360 colors selected from a continuous color wheel defined in the CIELAB color space with L = 70, a = 20, and b = 38, and radius = 60. The experimental procedure is depicted in Fig. 1.

Participants started the learning phase with a mouse click. During the learning phase, participants studied sequences of either two (low load) or four (high load) colored objects (with sequence length randomly intermixed across trials). Each colored object was presented on the screen center (objects’ size = 100 pixels) against a black background for 1 s. Object offset was followed by a 0.5 blank interstimulus interval before the next colored object was presented. Following each sequence of two or four objects, JOLs were solicited. The silhouette of each object was presented in light grey with a scale presented below it. The objects appeared in the same order as they were presented in the learning phase. Participants were instructed to rate how well they thought they would remember the object’s color in a delayed test at the very end of the experiment on a scale ranging from 1 (*not at all*) to 100 (*best memory possible*) by moving the mouse along the scale. Initially, a 1 appeared below the slider. This value changed immediately depending on where the participants moved the mouse cursor. There was no time restriction for giving the JOL. After giving the JOLs, an immediate (working memory) test followed. In the original sequence order, the silhouette of each object of the sequence appeared again on the screen center, and 1 s later a color wheel surrounded it. Participants used the mouse to select the color they remembered for the depicted object from the color wheel. When moving the mouse cursor along the wheel, the object was filled with the color at the current mouse position. When participants were satisfied with the selected color, they pressed the mouse button to confirm their response. Altogether, 100 objects were presented in each sequence-length condition (namely, 50 trials of Sequence-Length 2 and 25 trials of Sequence-Length 4). Trials with two and four objects were randomly intermixed, hence participants could not predict sequence length. Finally, at the end of the experiment, participants performed a delayed (long-term memory) test where they reproduced the color of each studied object in random order. The test procedure was the same as for the immediate recall task.

### Results

#### Sequence-length effect on recall and JOLs

We computed a measure of recall error for the immediate and delayed test by computing the absolute difference between the presented color and participants’ response. This measure can range from 0° (the correct color was chosen) to 180° (a color on the opposite side of the color wheel was chosen). We preregistered and performed a Bayesian ANOVA, with sequence length and test time point as predictors, participants as a random effect, and recall error as the predicted variable. All Bayesian ANOVAs reported here were conducted in R (R Core Team, 2018), using the default prior settings of the BayesFactor package (Morey et al., 2018). The results provided strong evidence in favor of an interaction between sequence length and test time point on recall error, BF_10_ = 262.43. We then performed two additional Bayesian *t* tests for paired samples to investigate the influence of sequence length on recall error separately for the immediate and delayed test. Besides reporting the Bayes factors in favor of the effect, we used the Bayesian Estimation Software (BEST) developed by Kruschke (2013) to get an estimate of the effect size *d* as well as its 95% highest density interval (HDI) reflecting the range of credible values for the effect size. Throughout this work, we report this effect size *d* for all Bayesian *t* tests for dependent samples.

**Fig. 2:**
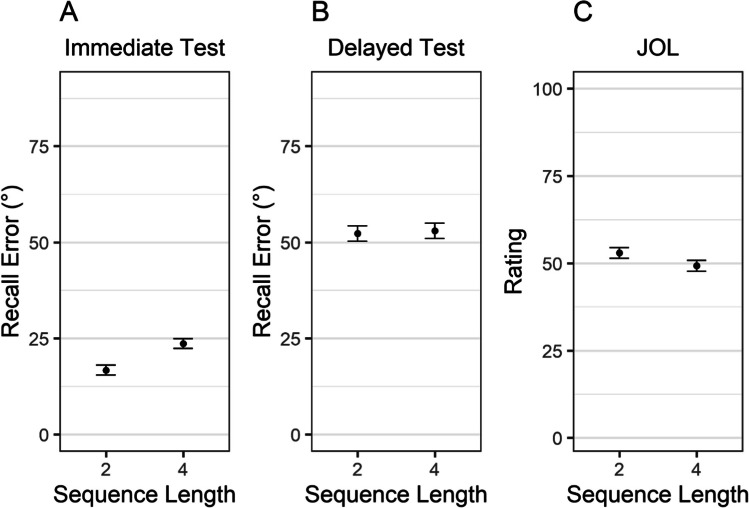
Recall error in the immediate (**A**) and delayed test (**B**) and judgments of learning (**C**) as a function of sequence length in Experiment 1. *Note.* Error bars represent 95% within-participant confidence intervals

As depicted in Fig. 2A, immediate recall error was lower for objects presented in sequences of two than four, BF_10_ = 7.87×10^5^, *d* = −1.4 [−1.93, −0.86]. As expected, this sequence-length effect was not found for the delayed test (see Fig. 2B), BF_10_ = 0.22, *d* = −0.09 [−0.46, 0.31]. Even though delayed performance was not influenced by sequence length, participants gave—as predicted—higher JOLs to objects learned in sequences with two compared with four objects (see Fig. 2C), BF_10_ = 19.78, *d* = 0.7 [0.23, 1.17].

#### Correlations between JOLs and immediate and delayed performance

**Fig. 3:**
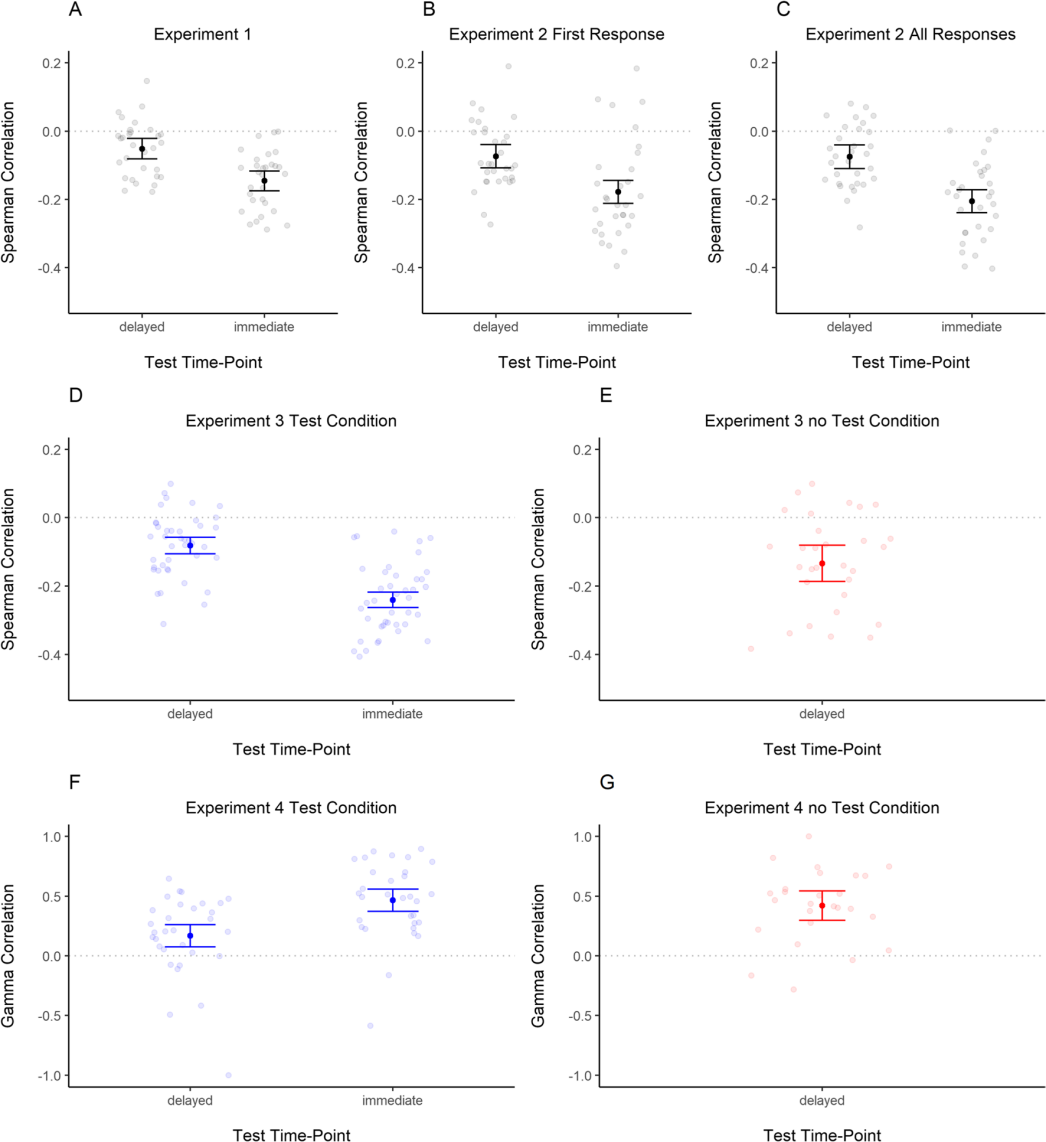
Correlations between judgments of learning and recall error in the delayed test and immediate test in each experiment. *Note*. Error bars represent 95% within-participant confidence intervals. Transparent dots represent the correlation coefficients of individual participants. Correlation coefficients were transformed to Fisher’s *z* scale prior to averaging them and computing confidence intervals. They were then retransformed to the *r* scale. Panels **A** to **E** present Spearman correlations. Panels **F** and **G** present Gamma correlations

For each participant, a correlation between JOLs and recall error in the delayed test was computed following our preregistered analysis plan. This correlation reflects the accuracy of their JOLs. Additionally, we computed the correlation between recall error in the immediate test and JOLs, reflecting how strongly JOLs are related to the people’s working memory representations. Given that both dependent measures were provided on a continuous scale, we used a parametric Spearman correlation method. The Spearman correlation was selected because recall error and JOLs were not normally distributed in our sample. The correlations were transformed to Fisher’s *z* scale before computing the average or performing Bayesian *t* tests. Note that because our memory measure is error, negative correlations should be expected (i.e., JOLs are higher when error is smaller). Figure 3A presents the individual and sample’s mean correlation. Despite reflecting people’s predictions for delayed memory performance, JOLs correlated higher with immediate than with delayed recall error (BF_10_ = 373, *d *= −0.85 [−1.32, −0.42]).

As preregistered, we implemented the confidence model proposed by van den Berg et al. (2017) but encountered unexpected problems.^2^ We will therefore not discuss this model further.

### Discussion

Experiment 1 provided first evidence for the use of working memory representations when making JOLs. Although sequence length did not affect delayed memory performance, participants gave higher JOLs when learning sequences of two than four objects, and JOLs were more correlated with immediate than delayed performance. Hence, the results of this experiment suggest that low JOL accuracy in predicting delayed performance does not invalidate the memory-strength theory. As shown in this experiment, it is possible that an experimental manipulation affects only immediate, but not delayed memory performance.

Two concerns can be raised regarding the procedure used in Experiment 1. First, there was a delay between studying the items and making JOLs. This delay was caused by the sequential presentation of the memoranda, followed by the sequential cueing of the objects for the JOL rating. This delay is important to consider as the literature shows that delaying JOLs can lead to more accurate predictions of long-term memory, known as the delayed JOL effect (Nelson & Dunlosky, 1991). The second concern that can be raised is that the delay systematically differed between the sequence length conditions. Because less objects were presented and cued in the Sequence-Length 2 condition as compared with the Sequence-Length 4 condition, the delay was shorter in the Sequence-Length 2 condition throughout the experiment. To address this possible confound and test whether we can replicate our findings when memory load is manipulated without introducing such a delay, we conducted Experiment 2.

## Experiment 2

In Experiment 2, we modified the experimental procedure to test whether the results of Experiment 1 can be replicated when JOLs are assessed immediately after presenting the colored objects. As in Experiment 1, we manipulated the working memory load by presenting two or four objects on the screen. However, in Experiment 2, the objects were presented simultaneously and JOLs were assessed right after the objects were shown. By implementing this modification, we reached two goals. First, for the very first JOL there was no delay between the end of the study phase and the JOL rating in both sequence-length conditions. Second, the delay between the study phase and the JOLs was generally shorter than in Experiment 1. We expected to still observe a sequence-length effect on JOLs, even for the very first rating.

### Method

#### Participants

The design consisted of a within-subjects manipulation of memory load (two vs. four objects simultaneously presented). Even though we used a different operationalization of the memory load than in Experiment 1, we expected the effects to be of similar size. Following the same considerations as in Experiment 1, we therefore decided to collect data of at least 30 participants. Overall, 31 participants were recruited from the platform Prolific and participated in this online experiment. Participants were between 18 and 35 years old, fluent in English, and had no visual impairment. In exchange for their participation, they received 6 GBP.

#### Materials and procedure

**Fig. 4:**
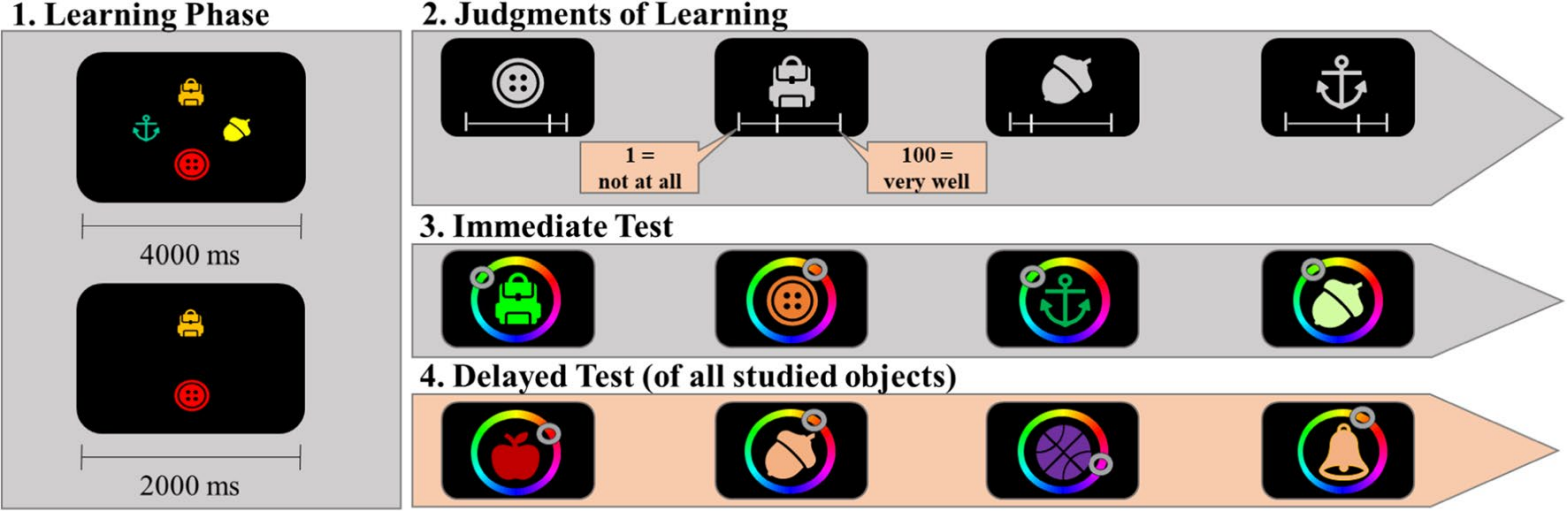
Illustration of the sequence of events in Experiment 2

The experiment was conducted online. The task was programmed using lab.js (Henninger et al., 2019), which is a HTML and JavaScript builder for constructing experiments online. The code for running the experiment is available in our OSF project. For the stimuli, we created a pool of 209 concrete objects. The procedure was the same as in Experiment 1—except for the following few changes: Instead of presenting a sequence of two versus four objects, two versus four objects were simultaneously presented on the screen as illustrated in Fig. 4. This manipulation will be referred to here as memory set size. In the Set-Size 4 condition, objects (100 pixels) appeared simultaneously at the top, right, bottom, and left position, 150 pixels away from the screen center. In the Set-Size 2 condition, objects appeared at the top and bottom (150 pixels away from the screen center). To make sure that all objects in both set-size conditions were encoded, the presentation duration was adapted to the number of objects presented. In the Set-Size 2 condition, the screen with the objects was shown for 2 seconds. In the Set-Size 4 condition, the screen was shown for 4 seconds. Immediately after the offset of the objects, the JOL phase started: objects of the trial were sequentially presented in the middle of the screen in light-grey color, one after the other, in random order for rating. Accordingly, there was no delay between the offset of the memoranda and the very first JOL rating in both set-size conditions. After the rating, immediate memory for all presented objects was probed in random order. The object appeared in the center of the screen surrounded by a color wheel (250 pixels away from the center). The color wheel was defined in the CIELAB color space as in Experiment 1 (with L = 70, a = 20, b = 38, and radius = 60). A minor change to the procedure of Experiment 1 was that at the immediate and delayed test, the color wheel appeared directly together with the silhouette of the objects (and not with a delay of 1 s).

As in Experiment 1, 200 objects were randomly selected for each participant to serve as memoranda in the experimental trials. Additionally, four objects were selected to serve for one practice trial. Overall, the experiment consisted of 50 trials of Set-Size 2, and 25 trials of Set-Size 4. Trials with two and four objects were randomly intermixed. As in Experiment 1, participants completed cycles consisting of a (1) learning phase followed by a (2) JOL phase, and (3) an immediate memory test. The experiment ended again with a delayed long-term memory test of all 200 objects.

### Results

In Experiment 2, we analyzed the data in two steps. In the first step, we included only the data of the very first object that was cued during the JOL phase. The results of these analyses are very insightful because solely for these observations the delay between the presentation of the objects and the assessment of the JOLs is the same for both set-size conditions. In the second step, we repeated the analyses for all objects of the trial. Including all objects did not affect the pattern of results. In the following, we therefore focus on the analyses of the very first object.

#### Set-size effect on recall and JOLs

**Fig. 5:**
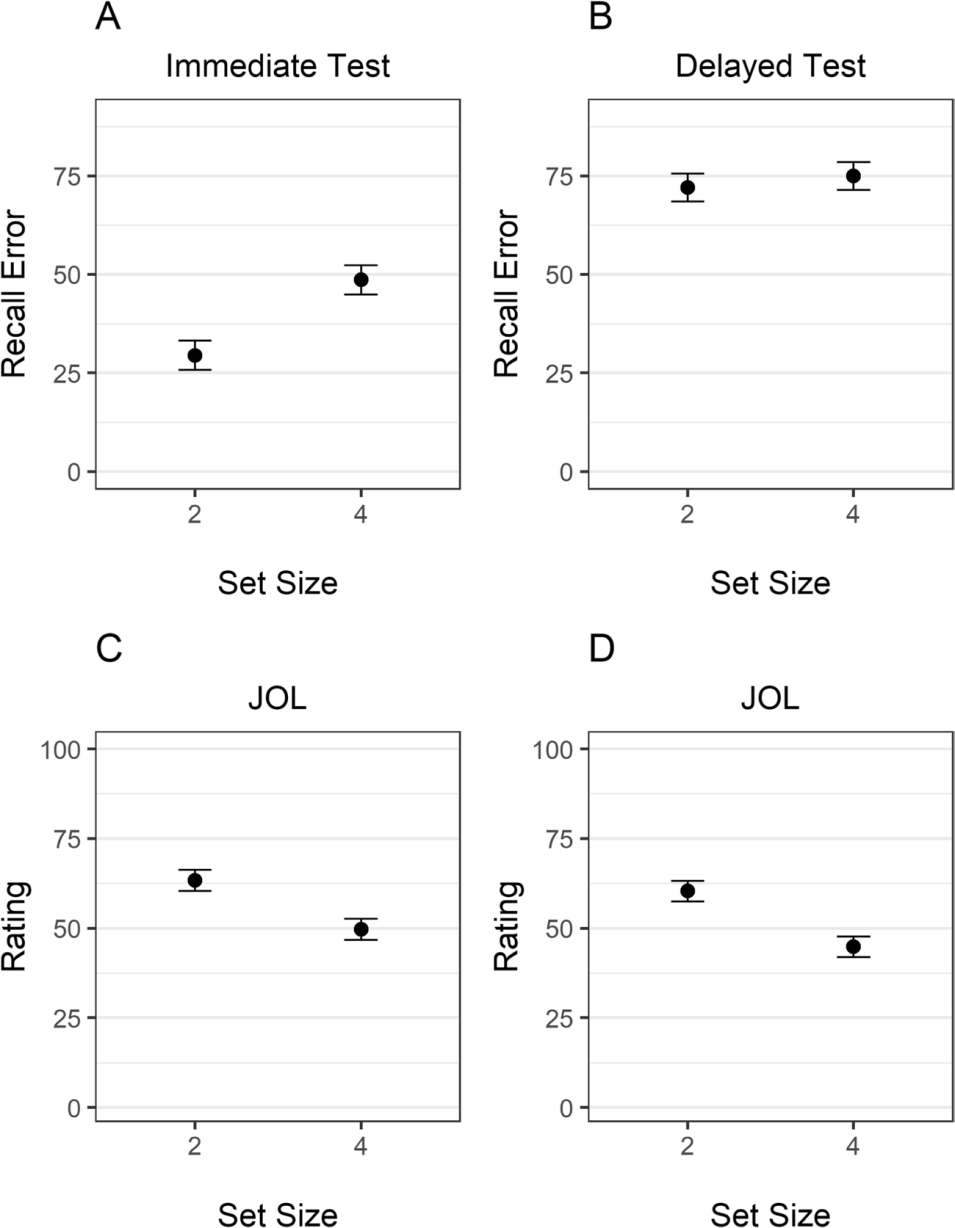
Recall error in the immediate (**A**) and delayed test (**B**) and judgements of learning for the first-rated object (**C**) and when considering all objects (**D**) as a function of set size in Experiment 2. *Note*. Error bars represent 95% within-participant confidence intervals

As in Experiment 1, we first performed a Bayesian ANOVA, with set size and test time point as predictors, participants as random effect, and recall error as the predicted variable. The results provided strong evidence in favor of an interaction between set size and test time point on recall error, BF_10_ = 4.80×10^5^, replicating the results of Experiment 1. We proceeded by conducting two *t* tests for paired samples in which we investigated the influence of set size on recall error separately for the immediate and delayed test. As in Experiment 1, immediate recall error was lower for the set-size two condition than set-size four condition, BF_10_ = 5.61×10^5^, *d* = −1.37 [−1.9, −0.85]. Again, this set-size effect was not found for the delayed test (see Fig. 5B), BF_10_= 0.37, *d* = −0.21 [−0.57, 0.17]. Consistent with our previous results and despite controlling for the temporal delay, participants gave higher JOLs to objects in the Set-Size 2 than the Set-Size 4 condition when considering the very first rating (see Fig. 5C), BF_10_ = 8.51×10^4^, *d* = 1.24 [0.74, 1.77]. This was also the case when we considered all ratings in a trial (see Fig. 5D), BF_10_ = 1.08×10^6^, *d* = 1.38 [0.87, 1.91].

#### Correlations between JOLs and immediate and delayed performance

To investigate the correlations between JOLs and immediate and delayed test performance, we proceeded as in Experiment 1. Figure 3B and C presents the individual and sample’s mean correlation with the first rating and all ratings, respectively. As in Experiment 1, JOLs correlated higher with immediate than with delayed recall error. This was the case for the object that was rated first, BF_10_ = 251, *d* = −0.83 [−1.26, −0.38], as well as when considering the ratings for all objects BF_10_ = 3.16×10^3^, *d* = −0.97 [−1.44, −0.53].

### Discussion

In contrast to Experiment 1, where objects were presented sequentially, in Experiment 2 we manipulated the memory load by simultaneously presenting either two or four objects on the screen. This allowed us to reduce the temporal delay between the presentation of the objects and the assessment of the JOLs. Critically, we were able to analyze the data of the very first object cued for rating for which there was no temporal delay between object presentation and JOL in both set size conditions.

The results were in line with the results of Experiment 1: In the immediate test, participants gave more accurate responses for objects in the Set-Size 2 as compared with the Set-Size 4 condition. However, the set-size manipulation did not affect participants’ performance in the delayed memory test. Crucially, participants gave higher JOLs for objects presented in the Set-Size 2 as compared with the Set-Size 4 condition. This was the case even for the very first rated object, where JOLs were assessed immediately after the objects’ offset. Thus, our results show that working memory load influences JOLs irrespective of the encoding mode (sequential or simultaneous), and therefore the delay of the rating cannot explain our findings. The lack of an effect of delay on JOLs here is not surprising since our delays were much shorter (2 or 4 seconds) than what is typically used to assess the delayed JOL effect (>1 min).

One concern that can be raised regarding the experimental procedure of Experiments 1 and 2 is that the inclusion of the immediate test artificially increased the salience of working memory representations. It is possible that this increased salience of the working memory test nudged participants into using working memory representations as a basis for JOLs. We addressed this possible limitation in Experiment 3.

## Experiment 3

In Experiment 3, we examined whether working memory representations are intuitively used to make JOLs by introducing a between-subject condition to the design of Experiment 1: While some participants made JOLs and then engaged in an immediate test (replicating Experiment 1), other participants solely made JOLs during the study phase. If JOLs also vary as a function of sequence length in a “no-test” condition, this would provide further evidence for the intuitive use of working memory representation as a basis for JOLs.

Besides including a “no-test” condition, Experiment 3 was designed to test whether JOL accuracy increases when information is retrieved from long-term memory. To attain this, we included a Sequence-Length 8 condition, which exceeds people’s working memory capacity hence requiring participants to retrieve information from long-term memory. We expect the correlation between immediate and delayed performance to be higher when eight objects are studied in one sequence (as compared with two and four). Consequently, JOL accuracy should also be higher in this condition. Inclusion of this Sequence-Length 8 condition could thus provide additional insights into why delayed JOLs are more accurate: after a delay, working memory representations are replaced and retrieval from long-term memory is enforced.

### Method

#### Participants

We preregistered (see https://osf.io/j78un/?view_only=None) to start data collection with a sample *N* = 60, because this experiment included two between subject conditions. Given our reliance on Bayesian inferences, we planned to increase sample size if we obtained ambiguous evidence (BF < 3) to reject or accept our hypotheses. Accordingly, 60 participants aged 18 to 35 years, without visual impairment were first recruited on the platform Prolific to take part in an online experiment in exchange for 6.25 GBP. Due to the random assignment of participants to the conditions, we obtained only 19 participants in one of the two between-participant conditions and 41 participants in the other one. Therefore, we collected further 11 participants for this condition, resulting in an overall sample of 71 participants (35 female, 35 male, one other). Only English native speakers were recruited to reassure the understanding of instructions. No participant had to be excluded based on the same preregistered exclusion criteria as applied in Experiment 1.

The design of the experiment consisted of a between-subjects manipulation of immediate memory test (test vs. no-test) and a within-subjects manipulation of sequence length (two, four, or eight). Participants were randomly assigned to one of the two between-subject conditions resulting in 41 participants in the test condition and 30 participants in the no-test condition. This sample size was sufficient to obtain substantial evidence in favor or against our hypothesis, and hence we stopped data collection.

#### Materials and procedure

The experiment was conducted online. As in Experiment 2, the task was programmed using lab.js (Henninger et al., 2019). We again used a pool of 209 concrete objects. For each participant, 192 objects were randomly selected to serve as memoranda in the experimental trials. Additionally, four objects were selected to serve for one practice trial. Overall, the experiment consisted of 32 trials of length two, 16 trials of length four, and 8 trials of length eight. Given that memory for all objects learned was tested, this equated the number of responses per design cell (i.e., 64). Trials with two, four, and eight objects were randomly intermixed, and hence participants could not anticipate the sequence length. As before, each object was randomly assigned one of 360 colors selected from a continuous color wheel defined in the CIELAB color space with L = 70, a = 20, and b = 38, and radius = 60 (Zhang & Luck, 2008).

As in Experiment 1 (see Fig. 1), participants completed cycles consisting of a (1) learning phase followed by a (2) JOL phase, and—in case they were in the “test” condition—(3) an immediate test phase. The experiment ended again with a delayed long-term memory test of all 192 objects. As in Experiment 2, the color wheel appeared directly together with the silhouette of the objects in the immediate and delayed test (and not with a delay of 1 s as in Experiment 1). Apart from this and the inclusion of a “Sequence-Length 8” condition and a “no-test” condition, the experimental procedure was kept the same as in Experiment 1.

Due to a programming error in Experiment 3, responses collected at the color wheel had an imprecision of ca. −3°. For example, if the participant wanted to click on the 90° angle, the registered response differed from the actual intended response by ca. −3°. Additionally, there was a range of 8° at the right side of the color wheel for which all responses were considered the same value. On the unrotated color wheel, this would be responses from 0 to 8°. However, given that the color-wheel was randomly rotated from trial to trial, this randomly affected any of the responses. Only 2.8% of the responses were given at this region, and hence there was only a minor bias created by it. Both errors were consequence of the creation of overlapping areas of interest for clicking when using the java-script program. We note, however, that this minor measurement imprecision did not substantially affect the results, and we replicated the results of Experiments 1 and 2.

### Results

#### Sequence-length effect on recall and JOLs

**Fig. 6:**
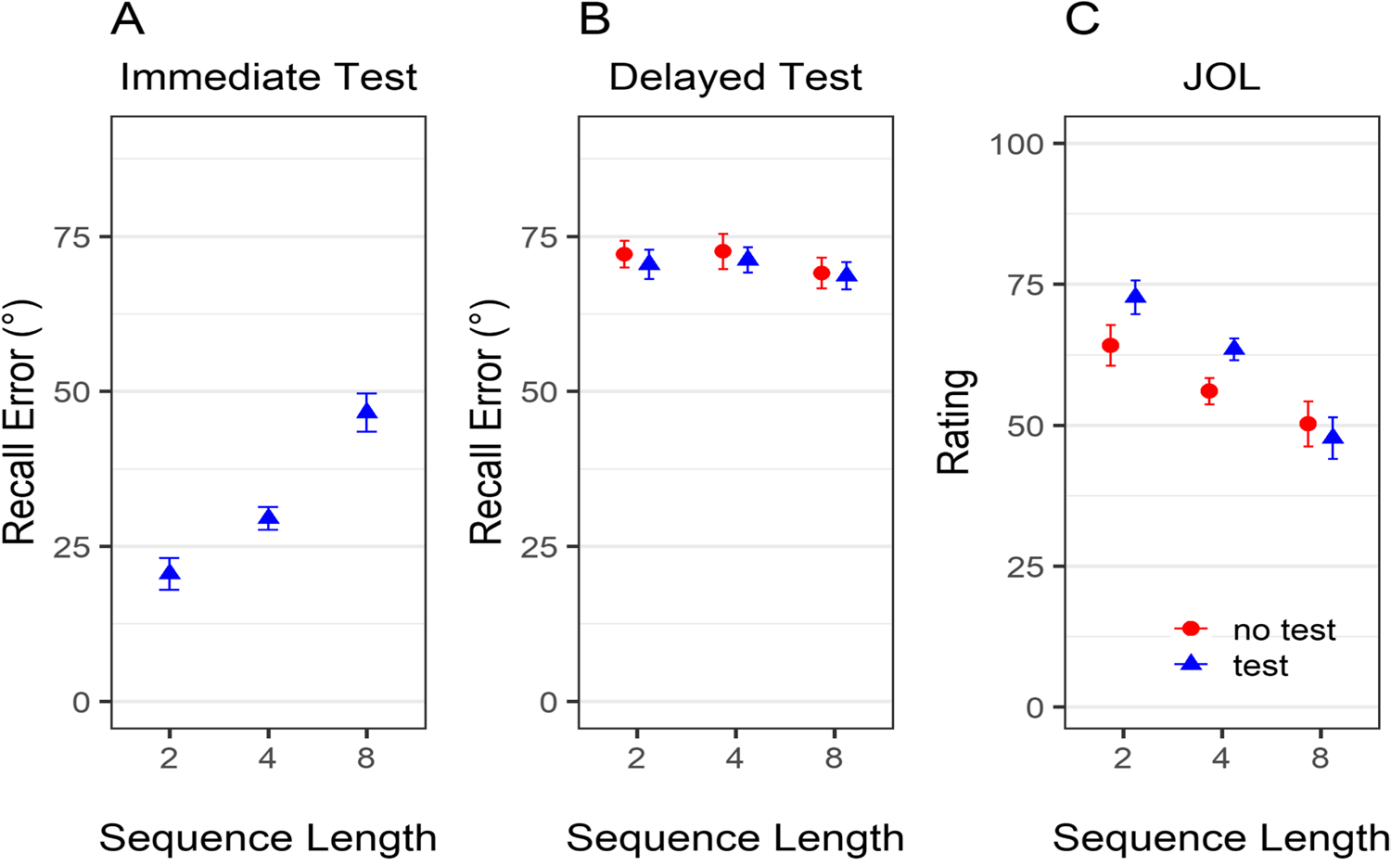
Recall error in immediate and delayed test and JOLs as a function of sequence length and test condition in Experiment 3. *Note*. Error bars represent 95% within-participant confidence intervals. Panel **A** depicts the data of participants in the test condition. Panels **B** and **C** depict the data of the full sample

Figure 6 shows the average recall error as function of sequence length, test time point, and test condition. As in Experiment 1, we preregistered and performed a Bayesian ANOVA, with sequence length and test time point as predictors, participant as a random effect, and recall error as dependent variable. For this analysis, we only used data of participants from whom we had observations for the immediate and delayed test (i.e., participants in the test condition). We found evidence for an interaction between sequence length and test time-point on recall error (see Fig. 6A–B), BF_10_ = 4.75×10^53^. We then computed two separate Bayesian ANOVA for the recall error data of the immediate test (with the data of the participants in the test condition) and delayed test (with the data of the whole sample). As in Experiments 1 and 2, the sequence-length manipulation affected performance in the immediate (BF_10_ = 1.68×10^141^), but not in the delayed test (BF_10_ = 0.008). A Bayesian hierarchical analysis yielded the same results (see Appendix  A).

In the next step, we conducted the preregistered analysis of participants’ JOLs as a function of sequence length and test condition (see Fig. 6C). This analysis included the data of the whole sample. The Bayesian ANOVA provided substantial evidence for an interaction between sequence length and test condition on JOLs, BF_10_ = 2.76×10^20^. Thus, sequence length affected JOLs differently depending on whether participants performed the immediate test or not. The same result was obtained using a Bayesian hierarchical analysis (see Appendix B). This interaction seems mainly related to sequence length having a larger effect on the test condition than in the no-test condition. Critically, our hypothesis was that sequence length affected JOL even in the absence of an immediate test. To test this prediction, we computed two additional Bayesian ANOVAs, separately for participants in the no-test and test condition. Here again, we found overwhelming evidence for an effect of sequence length on JOLs in both the no-test condition (BF_10_ = 9.12×10^43^) as well as the test condition (BF_10_ = 1.44×10^240^): JOLs were influenced by sequence length even in the no-test condition, although sequence length did not affect delayed performance.

#### Correlations between JOLs and immediate and delayed performance

As in Experiment 1, we computed correlations between JOLs and the recall error in the immediate test (with the data of participants in the test condition) and delayed test (for each group separately). Again, JOLs were more correlated with immediate than delayed performance (see Fig. 3D–E). We performed a Bayesian *t*-test for paired samples with data of the participants in the test condition. The results provided overwhelming evidence for this difference, BF_10_ = 1.64 ×10^9^, *d* = −1.51 [−1.99, −1.03]. There was ambiguous evidence for differences between the test and no-test groups regarding JOL accuracy in predicting LTM performance, BF_10_ = 1.09, *d* = −0.43 [−0.93, 0.09].

**Fig. 7:**
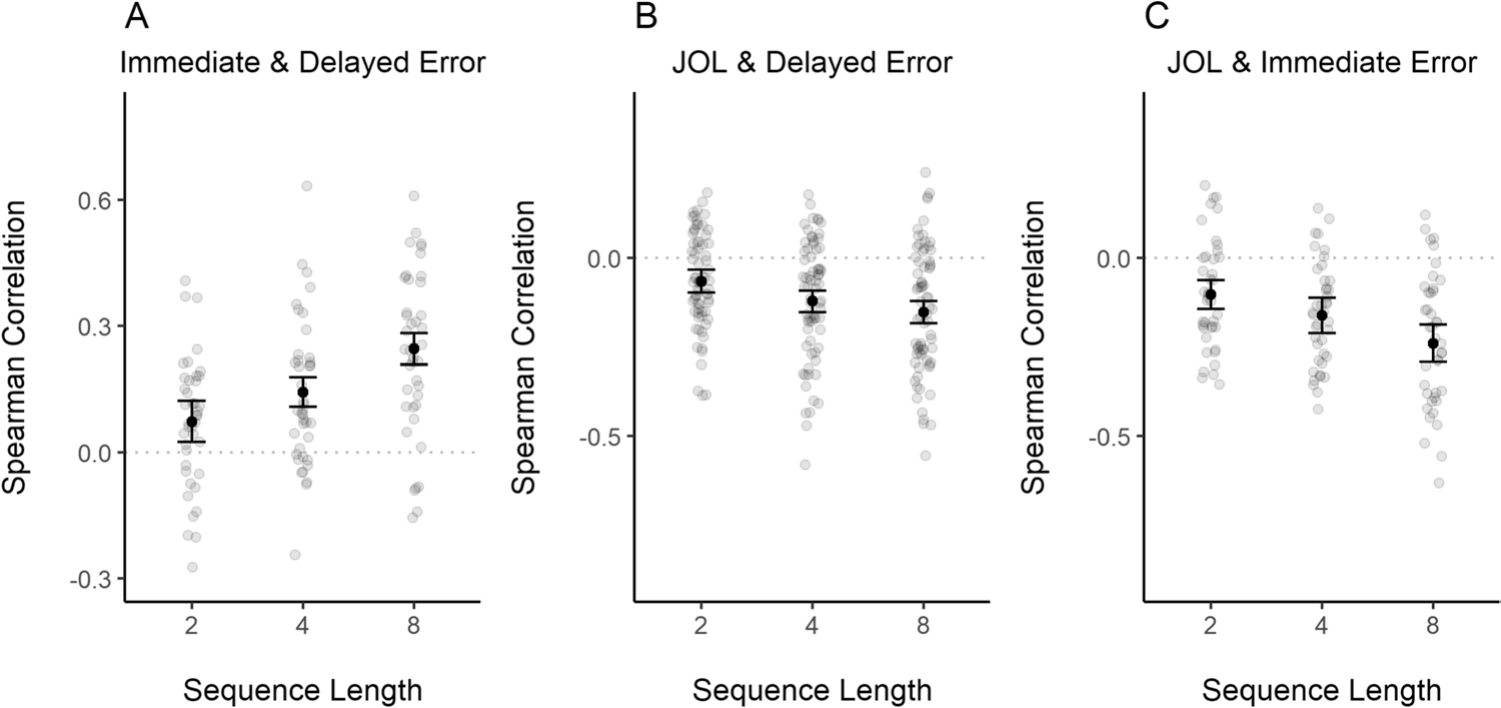
Correlation between immediate and delayed recall error (**A**), JOLs and delayed recall error (**B**), and JOLs and immediate recall error (**C**) as a function of sequence-length and type of correlation in Experiment 3. *Note*. Error bars represent 95% within-participant confidence intervals. Correlation coefficients were transformed to Fisher’s *z* scale prior to averaging them and computing confidence intervals. They were then retransformed to the *r* scale

We then computed the correlation between immediate and delayed recall error, JOLs and delayed recall error (JOLs’ accuracy) and JOLs and immediate recall error for each sequence-length condition (see Fig. 7).

Our prediction was that the correlation between immediate and delayed recall increases with increasing sequence length (Fig. 7A). This prediction is based on the assumption that participants have to retrieve the color from long-term memory in both the immediate and delayed test when the number of colored objects exceeds their working memory span. In contrast, when the number of colored objects is within participants’ working memory span, they retrieve the colors from working memory when engaging in an immediate test or making JOLs, but have to retrieve the colors from long-term memory when engaging in the delayed test leading to less accurate JOLs. Consistent with our prediction, the correlation between immediate and delayed performance for the test group was highest in the Sequence-Length 8 condition (see Fig. 7A). Our preregistered Bayesian modelling analysis led to the same result (see Appendix C).

In the next step, we examined the correlation between JOLs and delayed recall error, reflecting participants’ JOLs’ accuracy (see Fig. 7B). For this analysis, we used the data of the whole sample (i.e., the test and no-test condition combined). As predicted, JOLs’ accuracy varied with sequence length: Participants were most accurate in predicting their future performance in the Sequence-Length 8 condition, which exceeds average working memory span.

Lastly, we assessed the correlations between JOLs and immediate recall error in each sequence-length condition in the test group (Fig. 7C). Again, these correlations were higher with increasing sequence length. It is important to note that immediate recall error depends on the sequence-length condition: it is smallest in the Sequence-Length 2 and largest in the Sequence-Length 8 condition. By analyzing the data of each sequence length separately, we artificially restrict the variance of the immediate recall error in the shorter length conditions. Consequently, the correlation coefficients that are computed based on the immediate recall data of individual sequence-length conditions are decreased.

### Discussion

Experiment 3 corroborates the findings of Experiments 1 and 2: People intuitively use their current memory representations to make JOLs, even in the absence of an immediate test. Even though, the effect of the load manipulation on JOLs was stronger in the test condition, participants’ JOLs in the no-test condition also mimicked immediate memory performance speaking for the use of working memory representations even when working memory representations were not made relevant by an immediate test.

Additionally, we observed an increase in JOLs’ accuracy in the Sequence-Length 8 condition. This increase can be explained by the involvement of long-term memory when working memory span is exceeded.

A limitation of Experiments 1-3 is that both experiments used a paradigm and material that is novel to the literature on JOLs. It is therefore possible that the findings are specific to this experimental set-up and do not generalize to other materials. To test the generalizability of our conclusions, we performed Experiment 4 with verbal stimuli.

## Experiment 4

The goal of Experiment 4 was to test the working memory strength theory using a different, more commonly used task in the JOL literature—namely, a word-pair task. As in the previous experiments, we manipulated memory load by presenting participants with either two or four word-pairs for studying. Again, we compared participants’ working memory performance (assessed in one of the two between-subject conditions) with participants’ JOLs. Thus, Experiment 4 served as a replication of the previous experiments using verbal stimuli, and a task that has been commonly used to assess JOLs.

### Method

#### Participants

We started data collection with a sample *N* = 60. Given our reliance on Bayesian inferences, we planned to increase sample size if we obtained ambiguous evidence (BF < 3) to reject or accept our hypotheses. Sixty participants aged 18 to 35 years, without visual impairment were first recruited on the platform Prolific to take part in an online experiment for 2.40 GBP. Due to the randomization procedure, we obtained only 27 participants in one of the two between-participant conditions. We then collected further 3 participants for this condition, thus obtaining overall a sample of 63 participants (38 female, 25 male). Only English native speakers were recruited to reassure the understanding of the instructions.

The design of the experiment consisted of a between-subjects manipulation of immediate memory test (test vs. no-test) and a within-subjects manipulation of sequence length (two or four word pairs). Participants were randomly assigned to one of the two between-subject conditions resulting in 33 participants in the test condition and 30 participants in the no-test condition. This sample size was sufficient to obtain substantial evidence in favor or against our hypothesis, and hence we stopped data-collection.

#### Materials and procedure

The task was programmed using lab.js (Henninger et al., 2019) and was conducted online in the year 2022. For each participant, 124 words were randomly sampled from a pool of 594 English nouns and randomly paired into 62 word pairs. The 594 words were retrieved from the MRC Psycholinguistic data base (https://websites.psychology.uwa.edu.au/school/MRCDatabase/uwa_mrc.htm). Only highly frequent words with a length of four to five letters were selected. Fifty-six word pairs served as memoranda in the experiment; six word pairs served for two practice trials.

**Fig. 8:**
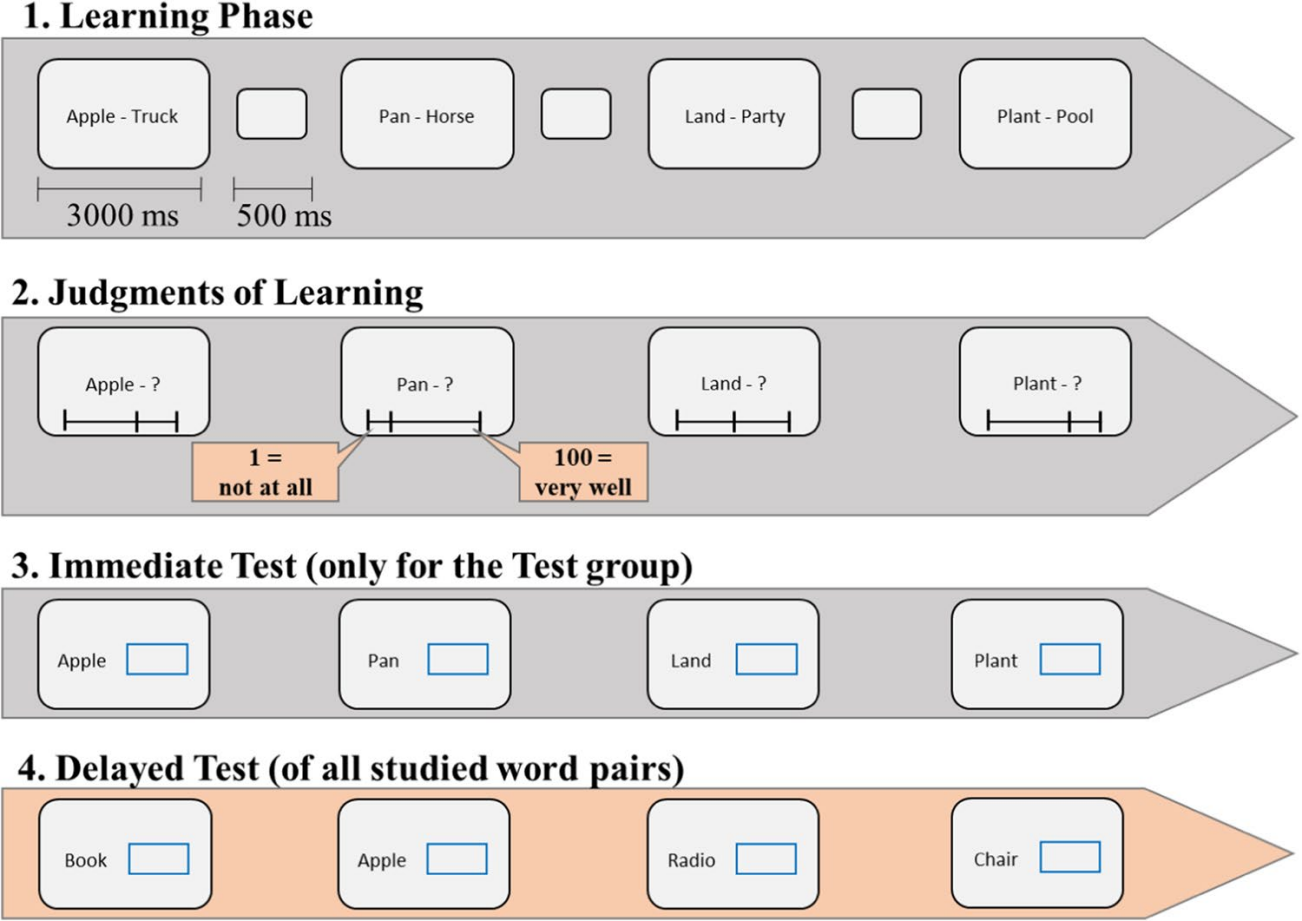
Illustration of the sequence of events in Experiment 4

Overall, participants studied 56 word pairs for a final test. As shown in Fig. 8, the word pairs were presented sequentially for 3 s in in the middle of the screen, separated by a dash (i.e., Apple–Truck). After 3 s, an interstimulus interval of 500 ms followed before the next word pair was presented. A sequence consisted either of two word pairs (low memory load) or four word pairs (high memory load), and sequences of two and four items were randomly intermixed. In total, the experiment consisted of 21 trials—14 trials of length two, seven trials of length four. Given that all word pairs were probed, this assured an equal number of responses in each design cell.

After the word pairs were presented, the first word of each pair appeared again on the screen with a question mark (i.e., Apple–?) and a slider underneath it. Participants were asked to indicate their likelihood of remembering the second word when cued with the first word in the final test. To give their response participants had to move the cursor along the slider. When moving the cursor, a number between 0 and 100 appeared below the slider depending on the cursor position. Participants had to click with the mouse when they were satisfied with their response. Pairs were probed in serial order.

After giving their JOLs, participants in the test-condition were again presented with the first word of each pair and had to recall the second word of the pair: a box appeared next to the first word enabling participants to type their response. Pairs were tested in serial order. Participants in the no-test condition were not asked to recall the words after they submitted their JOLs.

After learning all 56 word pairs, the delayed memory test started. In this test, word pairs were tested in random order. The first word of each pair appeared on the left side in the middle of the screen with a response box at the right. Participants were asked to type the second word of the pair and to confirm their response with the enter button.

Prior to the start of the experiment, participants performed two practice trials (one trial for each of the sequence-length conditions) to get familiar with the task procedure. These practice trials were excluded from the analyses and participants were informed that these words were not part of the list they should memorize for the final test.

### Results

#### Sequence-length effect on recall and JOLs

**Fig. 9:**
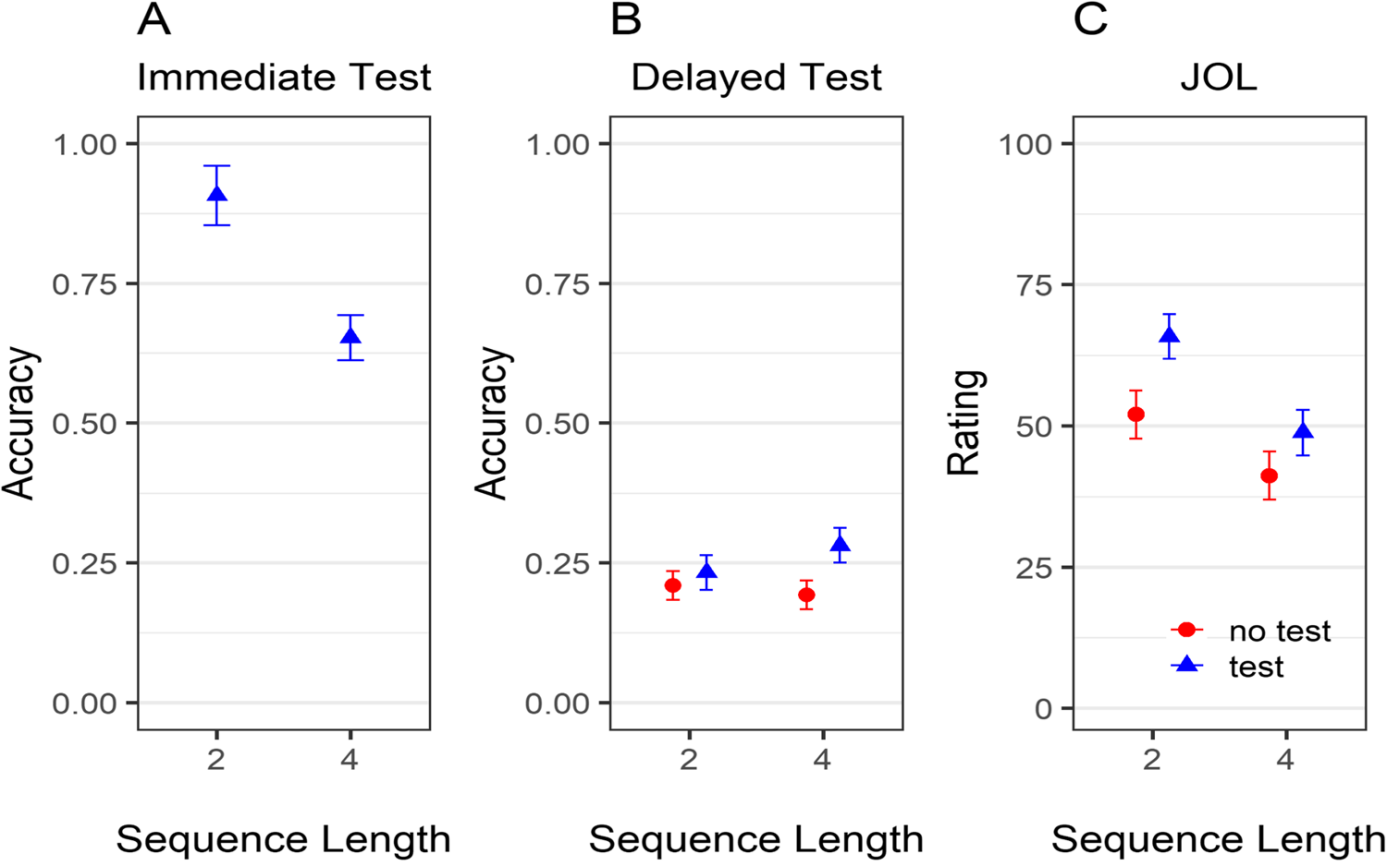
Accuracy in immediate and delayed test and JOLs as a function of sequence length and test condition in Experiment 3. *Note*. Error bars represent 95% within-participant confidence intervals. Panel **A** depicts the data of participants in the test condition. Panels **B** and **C** depict the data of the full sample

Figure 9 shows average recall accuracy as function of sequence length, test time point, and test condition in Experiment 4. To analyze the data, we first performed a Bayesian ANOVA, with sequence length and test time point as predictors, participant as a random effect, and accuracy as dependent variable. For this analysis, we only used data of participants from whom we had observations for the immediate and delayed test (i.e., participants in the test condition). We found evidence for an interaction between sequence length and test time point on accuracy (see Fig. 9A–B), BF_10_ = 6.15 × 10^27^. We then conducted two paired *t* tests assessing the sequence-length effect: one for the immediate recall and one for the delayed recall. There was overwhelming evidence for an effect of sequence length on immediate recall, BF_10_ = 1.69×10^5^, *d* = 1.19 [0.72, 1.66], but not in the delayed recall, BF_10_ = 0.63, *d* = −0.05 [−0.13, 0.02]. To further substantiate the lack of a sequence-length effect on the delayed recall, we also computed a Bayesian ANOVA with sequence-length and test condition as predictors, LTM accuracy as criteria and participant as random effect. There was evidence against inclusion of all predictors in the model, all BF_10_ < 0.26. For sequence length in particular, there was strong evidence against its effect on delayed memory performance in this analysis that considers the data of the whole sample (BF_10_ = 0.09).

In the next step, we conducted an analysis of participants’ JOLs as a function of sequence length and test condition (see Fig. 9C). For this analysis the data of the full sample was used. The Bayesian ANOVA provided evidence for an interaction between sequence length and test condition on JOLs, BF_10_ = 5.57. As in Experiment 3, sequence length affected JOLs differently depending on whether participants performed the immediate test or not, and this interaction seemed related to sequence length having a larger effect in the test condition compared with the no-test condition. We then computed two additional Bayesian *t* tests for paired samples with sequence length as predictor and JOL as criteria, separately for participants in the no-test and test condition. As in Experiment 3, we found substantial evidence for an effect of sequence length on JOLs in both the no-test condition (BF_10_ = 33.87, *d* = 0.67 [0.24, 1.07]) as well as the test condition (BF_10_ = 2.38×10^4^,* d* = 1.07 [0.62, 1.53]). Thus, JOLs were influenced by sequence length in both conditions, although the sequence length effect was bigger in the test condition.

#### Correlation between JOLs and performance in the immediate and delayed test

Given that memory responses in Experiment 4 were simply classified as correct and incorrect, and this is a binary variable, we computed a measure of rank correlations (aka Goodman–Kruskal’s Gamma correlation) to associate it with JOLs. We computed gamma correlations between participants’ JOLs and their performance in the immediate and delayed word-pair test. For these analyses, we excluded six participants who did not recall any word correctly in the delayed test and thus did not show any variance in performance making it impossible to compute correlations. Figure 3F–G depicts the correlations in the immediate and delayed test as a function of test condition. As shown in Fig. 3F, participants’ JOLs in the test condition correlated higher with the immediate as compared with the delayed test performance. A Bayesian *t* test for paired samples provided strong evidence for this difference, BF_10_ = 388.99, *d* = 0.84 [0.39, 1.27]. Unexpectedly, a Bayesian *t* test for independent samples showed that JOL accuracy in predicting LTM performance was higher in the no-test as compared with the test condition, BF_10_ = 8.07, *d* = 0.8 [0.17, 1.43], suggesting that the immediate test increased the salience of the working memory representations further reducing the accuracy of JOLs.

### Discussion

Experiment 4 replicated the findings of the first three experiments using a word-pair task. Consistent with the results from the visual color-object reproduction task, people also used their current working memory representations to make JOLs when learning verbal information. Even though this effect was bigger when working memory performance was assessed, it was also observed in the absence of a working memory test. Similar to the object color-reproduction task, working memory load had no impact on long-term recall replicating prior findings with word-pairs (Bartsch, Loaiza, & Oberauer, 2019a, 2019b). Thus, our findings are not specific to the color-reproduction task but generalize over different tasks and materials.

## General discussion

The present study showed that people used the strength of their working memory representations to make predictions regarding their future memory performance. This occurred although the fidelity of representations in working memory had little impact on how well participants could retrieve information in the final long-term memory test, and irrespectively of the inclusion of a working memory test, suggesting that people may naturally rely on their working memory to make future performance predictions. Our results are in line with several recent papers that have observed that people have direct metacognitive access to the contents of working memory (Forsberg et al., 2021; Honig et al., 2020; Krasnoff & Oberauer, 2022; Rademaker et al., 2012; Suchow et al., 2016; van den Berg et al., 2017; Yoo et al., 2021), that this access is already in place for children (Applin & Kibbe, 2020; Forsberg et al., 2021), and remains intact in old age (Mitchell & Cusack, 2018). Our findings highlight the importance of considering the role of working memory in metacognitive judgments in general, and JOLs in particular. Next, we will discuss the implication of our findings for theories of JOLs and limitations that should be addressed in future studies.

### JOL theories: Should direct access be reconsidered?

The theory that people make JOLs inferentially based solely on cues substituted the previously prevalent idea that JOLs are based on direct access to memory strength. In the present work, we argue that the memory-strength theory has been rejected prematurely because most prior studies did not measure or control for memory strength at the time point when JOLs are made, and hence they did not consider the role of working memory. Several recent papers have shown that people have access to the strength of their working memory representations therein (Forsberg et al., 2021; Honig et al., 2020; Krasnoff & Oberauer, 2022; Rademaker et al., 2012; Suchow et al., 2016; van den Berg et al., 2017; Yoo et al., 2021). Hence, it seems plausible that people use this information in making future memory predictions. Accordingly, we have proposed a revised version of the memory strength theory that considers that JOLs are guided by the strength of working memory representations as a viable candidate that should be considered. In four experiments, we revisited the memory-strength theory by manipulating and measuring working memory strength. Using both visual and verbal materials, we showed that JOLs follow the same pattern as people’s memory performance at the time point when JOLs are made. Throughout the experiments, our memory load manipulation affected working memory performance and likewise people’s JOLs without affecting their long-term memory performance. Overall, these results show that the inaccuracy of JOLs—that was often taken as evidence for the use of cues—does not discard the memory-strength theory. On the contrary, part of the inaccuracy of JOLs can be explained by working memory traces not being always predictive of long-term memory performance.

The memory-strength theory can also explain one of the best-replicated findings in the field of metamemory, the delayed-JOL effect. In Experiment 3, we showed that JOLs become more accurate when information must be retrieved from long-term memory. This was the case when sequence-length was of eight items, and hence participants could not keep all items in their working memory. In contrast, when JOLs are made immediately after studying, and the amount of information is within working memory span, working memory representations are used to predict future performance. Because working memory representations are not always correlated with future memory performance, immediate JOLs are less accurate. This explanation of the delayed-JOL effect is in line with the monitoring dual-memories hypothesis (Nelson & Dunlosky, 1991). Crucially, direct access to the two representations in the two different memory systems might be sufficient to explain JOLs with no need to assume further inferential processes.

One observation that we made in Experiments 3 and 4 was that the presence of an immediate test increased the effect of working memory load on JOLs. This finding is in line with prior work that suggests that people do not always need to retrieve information to make a JOL (Son & Metcalfe, 2005; Tauber et al., 2015). Thus, whereas people rely on working memory consistently when they know they will be asked to retrieve working memory representations in an immediate test, they use them to a lesser extent when immediate performance is not assessed. Given that retrieving the information from working memory on each trial requires some effort, it is possible that participants avoided this effort on some trials of the no-test condition and just selected a value in the middle of the scale. This would also be consistent with our observations that values around 50 were frequently selected in this condition, especially in Experiment 4.

Finally, one recent study from our lab (Krasnoff & Overkott, 2022) also provided evidence for the role of working memory strength for JOLs. It is often observed that people prefer blocked than interleaved learning, and that they provide higher JOLs to the former than the later even though long-term learning is better for the interleaved learning condition. This has been considered a metacognitive illusion. This study showed that JOLs for blocked learning are higher because this condition is associated with higher working memory strength compared with interleaved learning. When the working memory advantage of blocked over interleaved learning was experimentally removed, JOLs were equal across conditions. These results are fully consistent with the findings of the present study, and further highlight the importance of considering working memory strength as the basis for JOLs.

### Study limitations

Although our findings were overall in line with the revised memory-strength theory, it is important to note that they do not discard the cue-utilization theory that could explain our findings with different mechanisms. An alternative cue-based explanation for our findings, for instance, is that people possess explicit beliefs regarding how memory load influences their memory performance (e.g., “When I study many objects, my memory for the objects’ colors is weaker”) and that those beliefs guided participants’ JOLs. Instead of accessing their working memory representations, participants might have used sequence length or memory set size as a cue to infer their likelihood of future recall. This explanation, however, fails to account for the correlations between JOLs and recall error that we found within each sequence-length condition (see Experiment 3). If people just used the sequence length as a cue for their JOLs, they would have assigned the same JOL to all objects of a sequence-length condition. In contrast, the correlation between JOLs and recall error within each sequence-length condition implies that people are sensitive to variations in working memory strength to make their predictions. In addition, the use of such a belief-based cue fails to explain why JOLs were more accurate when the number of information exceeded working memory span (in Experiment 3).

An alternative explanation for our findings is the use of experience-based cues, such as retrieval fluency (Benjamin et al., 1998; Koriat & Ma’ayan, 2005). In contrast to directly accessing the memory strength for the to-be judged item, people might translate the ease they feel when retrieving it into a JOL. The drawback of this experience-based cue explanation, however, is that retrieval fluency is hardly dissociable from actual memory strength. Empirically, retrieval fluency is commonly measured by retrieval success or latencies. These measures, however, are highly correlated with memory strength and retrieval success is normally used to assess the latter. If we assume that retrieval fluency is the only cue people use when there is variation in memory strength, the cue-utilization approach will make the same predictions as the memory-strength theory for JOLs: JOLs will vary with the memory strength for the to-be judged items. Future research is needed to dissociate retrieval fluency from memory strength and to show that the additional assumption about the use of retrieval fluency instead of direct access to memory is justified. The present findings, however, can be well accommodated by the memory-strength theory and without the additional assumption of those inferential processes.

It is also worth noting some limits on the generalizability of our findings. Similar to most previous studies on JOLs, we recruited a student sample to participate in Experiment 1. In Experiments 2–4, we conducted the study online using Prolific and recruited a sample of young adults. Our findings thus apply to young adults that have a relatively high level of education. Future studies are needed to evaluate if our results hold for people with different age ranges, and a more varied education background and hence possibly less exposition to demands to monitor their ongoing learning.

## Conclusion

The results of the four experiments with educated young adults showed that JOLs varied with the load of information in working memory, although this was not predictive of future memory performance. The cue-utilization approach can accommodate these findings by the use of belief-based cues regarding the effect of memory load or by the use of retrieval fluency as a cue for JOLs. Both of these explanations have, however, limitations. The revised memory-strength theory proposed here, in contrast, can explain these findings by the use of working memory strength as a basis for JOLs. One major advantage of the revised memory-strength theory is that it is parsimonious and makes strong predictions that are easy to test. Finding that JOLs are not related to memory strength in a situation in which memory strength varies would provide conclusive evidence against the memory-strength theory (see also Krasnoff & Overkott, 2022). In contrast, the cue-utilization theory is hard to falsify. This is because it does not make strong predictions about which cues are utilized in which situations. Consequently, even finding that JOLs do not vary with the manipulation of a cue will not invalidate the cue-utilization approach. Because of the simplicity of the revised memory-strength theory as well as the evidence provided in this study, we therefore suggest that it should be further considered as a more parsimonious, alternative explanation for JOLs.

## Figures and Tables

**Fig. 10:**
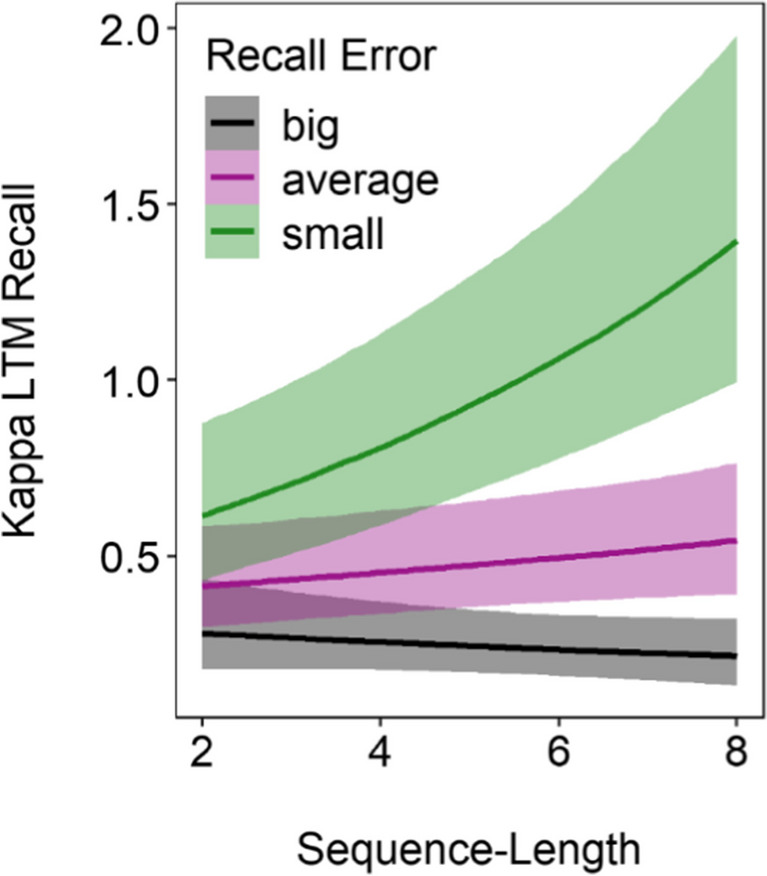
Prediction for Kappa (memory precision) as a function of sequence length and immediate recall error for the data of Experiment 3

## Appendix A

### Bayesian model predicting test performance (Kappa) by sequence-length and test time-point in Experiment 3

We estimated a Bayesian Model using the R-package brms (Bürkner, 2017, 2018). In this model, we predicted test performance in Experiment 3 with the continuous predictor sequence length (centered on Length 2) and the factor test time point having as a reference the immediate memory condition assumed to assess WM performance. Because the distribution of recall error is skewed, we did not model the recall error directly, but we modelled the deviance of the given response from the correct color. The deviance distribution is best described by a von Mises (normal distribution for circular space). As most responses are close to the correct response, this distribution peaks at a deviance of zero. The width of the von Mises distribution is captured by the precision parameter, Kappa. In our model, we predicted Kappa by the main effects of sequence length and test time point and their interaction. Originally, we planned to allow for a nonlinear relation of sequence length and deviance, but nonlinear parameters were not allowed for a von Mises family; hence, we fitted a standard linear model with four chains, each with 2,000 iterations (1,000 iterations were discarded as burn-in). Parameters converged as indicated by the R-hat values below 1.05. Table 2 summarizes the results.

**Table 2:** Population-level estimates for the effects of sequence-length, test time point, and their interaction on memory precision (Kappa) in Experiment 3 (values are reported on a logarithmic scale)

	Posterior estimate	Posterior estimate error	Lower 95% CI	Upper 95% CI
Sequence length	0.02	0.02	−0.02	0.06
Test time point	**2.49**	**0.16**	**2.16**	**2.81**
Sequence Length × Test Time Point	**−0.24**	**0.02**	**−0.29**	**−0.2**

CI = credible interval. Credible parameter values (i.e., for which zero is not included in the credible interval) are presented in boldface

The results support an interaction of test time point and sequence length on Kappa. Whereas test time point had a credible effect on Kappa, we did not find a main effect of sequence length. The supplementary materials file presents the model’s prediction against the data (i.e., a posterior predictive plot) showing that the model captured the data well.

## Appendix B

### Bayesian model predicting JOLs by sequence length and test condition in Experiment 3

We explored whether JOLs are influenced by the test condition and sequence length by estimating a Bayesian model in brms. To model participants’ JOLs, we used the zero-or-one inflated beta regression (ZOIB), a method to analyze data from continuous response scales. The main assumption of ZOIB is that the observed distribution of scale-response data evolves from two underlying distributions: A binomial distribution (participants choose either the highest or the lowest value of the scale) and a beta distribution (participants choose values in the whole range of the scale). The parameter phi caputers the precision of the beta distribution, the parameter zoi reflects the probability to be in the binomial distribution, and the parameter coi reflects the proportion of responses in the upper end of the scale. In our model, we predicted average JOLs as well as all three free parameter by sequence length, test condition, and their interaction. To apply this model, we first linearly transformed the JOLs into a scale ranging from 0 to 1. The variable sequence length was again centered on Length 2. We fitted the model with 4 chains, each with 10000 iterations (5000 iterations were discarded as burn-in). Parameters converged as indicated by all R-hat values being below 1.05. There was one divergent transition after warm-up.

Table 3 depicts the model’s population level estimates of the effects of sequence length, test condition, and the interaction of sequence length and test condition on average JOLs (see Supplementary Materials File for a comprehensive overview of effects in all parameters). The ZOIB results replicated the findings from the Bayesian ANOVA in Experiment 3: There was an interaction of sequence length and test time point on JOL and likewise two main effects of test time point and sequence length on JOLs.

**Table 3:** Population-level estimates for the effects of sequence length, test time point, and their interaction on JOLs (values are reported on a logit scale)

	Posterior estimate	Posterior estimate error	Lower 95% CI	Upper 95% CI
Sequence length	**−0.09**	**0.02**	**−0.13**	**−0.04**
Test time point	**0.65**	**0.25**	**0.16**	**1.15**
Sequence Length*Test Time Point	**−0.11**	**0.03**	**−0.17**	**−0.05**

CI = credible interval. Credible parameter values (i.e., for which zero is not included in the credible interval) are presented in boldface

## Appendix C

### Bayesian model predicting test performance (Kappa) in delayed test by sequence length and recall error in immediate test in Experiment 3

We estimated a Bayesian mixed-effects model in brms, with immediate recall error and sequence length as two continuous predictors and delayed recall error as dependent variable in Experiment 3. Statistically, this prediction should be reflected in the interaction between sequence length and immediate recall error on delayed recall error. Instead of predicting the recall error directly, we again used the von Mises distribution and modeled the precision parameter, Kappa. We fitted the model with four chains, each with 2,000 iterations (1,000 iterations were discarded as burn-in). Parameters converged as indicated by all R-hat values being below 1.05. Fig. 10 depicts the models’ predictions.

As expected, the model predicts an interaction between immediate recall error and sequence length on the precision of recall in the LTM test (see Table 4 and Fig. 10). At a Sequence-Length 8, the immediate recall error is more predictive for the delayed recall Kappa than at a Sequence-Length 2. In other words, when participants perform well in the immediate test in the Sequence-Length 8 condition, they are more likely to perform well on that object in the delayed test. In contrast, performing well on an object in the immediate test in a Sequence-Length 2 condition is weakly associated with better performance in the delayed test. Thus, the modelling result confirms our prediction: immediate and delayed recall error are higher correlated in the Sequence-Length 8 condition, supporting our assumption that in this set-size participants are more likely to be retrieving information from LTM.

**Table 4:** Population-level estimates for the effects of sequence length, immediate recall error, and their interaction on memory precision (Kappa) in the delayed test of Experiment 3 (values are reported on a logarithmic scale)

	Posterior estimate	Posterior estimate error	Lower 95% CI	Upper 95% CI
Sequence length	**0.12**	**0.02**	**0.08**	**0.16**
Immediate recall error	**−0.58**	**0.16**	**−0.93**	**−0.28**
Sequence Length × Immediate Recall Error	**−0.13**	**0.04**	**−0.21**	**−0.05**

CI = credible interval. Credible parameter values (i.e., for which zero is not included in the credible interval) are presented in boldface

## Footnotes

1 In some studies, JOLs are assessed after a temporal delay.

2 Before fitting the model to our JOL data, we first aimed to replicate the original findings of van den Berg and colleagues (2017) by reanalyzing confidence data collected in our lab (Krasnoff & Oberauer, 2022). In contrast to the original paper, in our replication the model failed to accurately predict participants’ confidence ratings. We therefore decided that fitting the model to JOL data would not be appropriate.
